# Au-Nanomaterials as a Superior Choice for Near-Infrared Photothermal Therapy

**DOI:** 10.3390/molecules191220580

**Published:** 2014-12-09

**Authors:** Fahmida Jabeen, Muhammad Najam-ul-Haq, Rabia Javeed, Christian W. Huck, Guenther K. Bonn

**Affiliations:** 1 Division of Analytical Chemistry, Institute of Chemical Sciences, Bahauddin Zakariya University, Multan 60800, Pakistan; E-Mails: fahmida-09-07@hotmail.com (F.J.); rabiapuic@gmail.com (R.J.); 2 Institute of Analytical Chemistry and Radiochemistry, Leopold-Franzens University, Innrain 80-82. Innsbruck 6020, Austria; E-Mails: christian.w.huck@uibk.ac.at (C.W.H.); guenther.bonn@uibk.ac.at (G.K.B.)

**Keywords:** near-infrared spectroscopy, nanoparticles/materials, therapy monitoring

## Abstract

Photothermal therapy (PPT) is a platform to fight cancer by using multiplexed interactive plasmonic nanomaterials as probes in combination with the excellent therapeutic performance of near-infrared (NIR) light. With recent rapid developments in optics and nanotechnology, plasmonic materials have potential in cancer diagnosis and treatment, but there are some concerns regarding their clinical use. The primary concerns include the design of plasmonic nanomaterials which are taken up by the tissues, perform their function and then clear out from the body. Gold nanoparticles (Au NPs) can be developed in different morphologies and functionalized to assist the photothermal therapy in a way that they have clinical value. This review outlines the diverse Au morphologies, their distinctive characteristics, concerns and limitations to provide an idea of the requirements in the field of NIR-based therapeutics.

## 1. Photothermal Therapy (PTT) and Nanoparticles

Photothermal therapy is a strong candidate in cancer treatments and therapies as it induces targeted heat destruction of tumor regions. For clinical therapy, NIR lasers are selected because of their higher penetration in human tissue resulting in minimal damage [1]. A bio-nanoporobe with multiple functionalities is capable of absorbing optical energy and converting it to heat, which leads to the idea of coupling optical lasers with nanotechnology [2]. Advancements in oncology treatment highlight irradiation of plasmonic nanoparticles (PNPs) via thermal ablation. The tendency of human tissue to transmit electromagnetic radiation in the specific near-infrared region helps to manipulate the properties of PNPs in accordance with the temperature required for tumor tissue therapy [3]. As a result research has been done to increase the absorption efficiency of PNPs in the NIR region [4]. Different photothermal materials capable of enhanced NIR absorption because of their non-invasive optical penetration are reported, among which the most popular are carbon nanotubes (CNT) [5], Au- based nanomaterials [6] and composites [7,8]. These materials also have potential for cellular and *in vivo* imaging when coupled to magnetic resonance imaging and microscopic/spectroscopic techniques [9,10].

The selection criteria for PNPs adopted for PTT depend on four characteristics which can be summarized as follows: (a) the capability to absorb in the NIR region (*i.e.*, 700–1,000 nm); (b) ensuring the size of the nanoparticles is less than 100 nm, allowing maximum absorption by the tissue; (c) enhanced absorption cross-section and (d) reduced toxicity with better biocompatibility [11,12]. The challenge faced by PNPs is their efficient delivery to target sites and penetration into the affected areas depends on their size and composition [13]. Also changes in the morphology (size and shape) of nanoparticles induce drift in the absorption to higher wavelengths required for cytotoxicity to kill the cancer cells [14]. This demands new economical routes for the fabrication of nanomaterials, reproducibility of photothermal efficiency, control of toxicity and eventual commercialization [15].

The best explored plasmonic material is gold (Au) because of its biocompatibility, low cytotoxicity, tendency to absorb in the NIR region due to plasmon resonance, excellent photostability, optical-thermal conversion effectiveness and availability in various morphologies [16]. Au-based nano-materials induce combined oscillation of conduction electrons upon optical excitation [17]. Au nanoparticles (~40 nm) convert optical irradiation (514 nm) to heat energy capable of killing cancer cells. Such tuned frequencies can be established for Au-nanomaterials in the NIR region, opening up the therapeutic window for better clinical oncology [18]. Gold nanoshells [19] and nanorods [20] are promising PNPs adopted for cancer therapy because of their enhanced optical absorption in the near-IR region. Gold nanomaterials also absorb higher energy X-rays, thus making them efficient CT imaging contrast agents, and suggesting a promising future for Au-nanomaterials in theranostics [21].

Spherical Au-nanoparticles can be used by controlling the size and attaining the desired NIR absorption [22]. They can be aggregated or allowed to self-assemble for absorption at longer wavelengths, however only the NIR absorption is not a sufficient criterion to conclude if a material is a good PTT agent. They must not only be transported to the target site but also cleared out from the body once the therapy is complete. Aggregated Au-NPs suffer from low disintegration which hinders their removal from the organs and prolongs their presence in the body which may eventually cause tissue damage or metal toxicity [23].

**Figure 1:**
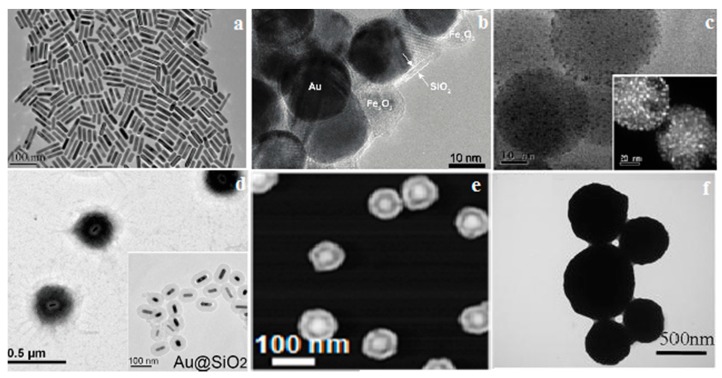
Morphology of Au-nanoparticles: (**a**) TEM image of Au-nanorods (GNRs) (**b**) High-resolution TEM image of Au/Fe_2_O_3_ nanoparticles coated with amorphous SiO_2_ shells, (**c**) TEM of GSS nanoparticles showing the speckled silica surface; inset z-contrast digital TEM, (**d**) TEM images of nanocomposite and Au@SiO_2_ (inset) (**e**) SEM image of nanomatryoshkas and (**f**) TEM image of self-assembly structured Au-nanoparticles. (reproduced from [25] (a), [26] (b), [27] (c), [22] (d), [28] (e), [29] (f) with permission).

Research based on Au nanomaterials is flourishing, however it lacks a commercial aspect [24]. Small spherical Au nanoparticles exhibit poor NIR absorption which lowers their efficiency for PTT, therefore morphologies like nanoaggregates, nanoshells, nanorods and nanomatryoshkas with polymeric or inorganic coatings are being introduced (Figure 1). 

This review presents the current Au-morphologies used as PNP materials for photothermal therapy coupled with NIR lasers. Table 1 summarizes the unique characteristics, advantages and limitations of Au-based nanomaterials. 

## 2. Hybrid Superparamagnetic Au-Nanoaggregates

Nanomaterials with super-paramagnetic behavior are used as bio-nanoprobes [30]. Such nano-materials can be controlled towards the specific target by applying an external field, which is especially beneficial in the case of tumors. In addition, magnetic hyperthermia affects the therapeutic action which subsequently increases the temperature when external magnetism is applied [31]. 

To make use of these properties, hybrid plasmonic superparamagnetic gold aggregates (50–100 nm) are fabricated using the flame aerosol method [26]. Gold nanoshells are not preferred in this area because their large sizes hamper their biodistribution, absorption and tissue uptake. Hybrid Au-aggregates are synthesized in single step process where gold and iron oxide nanoparticles are coated with amorphous SiO_2_. The amorphous silica coating (>1.4 nm) provides versatile surface chemistry with increased hydrophilicity [32] and enhances the compatibility of nanoaggregates to interact with the biological environment. 

**Table 1:** Characteristics, advantages and limitations of Au-nanomaterials.

Au-Nanomaterials	Characteristics	Advantages	Limitations
Gold Nanorods	Temperatures can be reached using a high powered LED device	Increase NIR light penetration Lack perfusion *in vivo* models Ablative affective accumulation in tumor tissues No change in morphology Better heating capability	Poor bio-distribution and clearance within the body due to higher GNRs concentration and power density for *in vivo* use
Silica-Coated Au/Fe_2_O_3_ Nanoaggregates	Tuning of Au inter-particle distance for better plasmonic activity	Better dispersion due to silica shell Effective optical absorption Available for MRI imaging Stable as compared to nanoshells and nanorods	Altered sensitivity dependent on different tissues to heat exposure Systematic investigation on the pharmacokinetics Require surface bio-functionalization
Luminescent gold speckled silica (GSS) nanoparticles	Comprised of GRAS materials Discontinuous and random deposits of nanogold on silica surface	Size tunability fluorescence, and visible-NIR broad extinction spectra Prone to aggregation Ability to traffic to the perinuclear region Monitoring uptake, internalization, localization, tumor penetration and bio-distribution Real time non-invasive imaging and therapy	Dependent aggregation on the amount of gold used in the reaction Caused microemulsion instability at high concentrations
Thermoresponsive polymer encapsulated Au-nanorods PNIPAM-Au@SiO_2_	Simultaneously deliver heat and anticancer drugs	In vivo thermo-responsive behavior Minimal cytotoxicity High biocompatibility	Temperature and pH-dependent size of nanoparticle size Half-life might not be sufficient for their effective accumulation in tumor
Amphiphilic mixed polymers grafted gold nanoparitcles	Distinct chemical property from amphiphilic polymers, which endowed the Au NPs ability to self-assembly •Potential CT contrast agent	Improved thiolation method Safe Low toxic reagent with good biodegradability	Could not be totally destroyed under NIR irradiation Can be decomposed due to hydrolysis of hydrophobic PCL *in vivo* Reduced disintegration in cell affecting clearance from tissue
Au nanomatryoshkas (Au/SiO_2_/Au)	Controlled silica thickness (oversized silica layer on Au core followed by controlled etch-back of the silica layer)	Enhanced permeability Biocompatible High efficiency	No statistical difference in performance of nanomatryoshks and nanoshells

The key point is to use SiO_2_ to tune the Au inter-particle distance, which affects the optical absorption in the NIR region [33]. The encapsulating SiO_2_ shell is used as a dielectric spacer, so the critical step is the fine tuning of the silica coating to obtain the desired photothermal therapy effects [34]. The presence of the SiO_2_ shell enhances the biodistribution [35] and avoids reshaping of the Au particles during laser irradiation [36]. Nanoaggregates are investigated as magnetic resonance imaging (MRI) agents using relaxivity measurements and efficient PTT agents due to the induced cytotoxicity in human breast cancer cells by four minutes of NIR laser exposure. The method adopted for coating (gas phase by swirl injection) provides impurity-free nanoaggregates shelled in silica. It is also important that the silica does not modify the inherent properties of the core materials like pure TiO_2_ [37], Ag [38] and Fe_2_O_3_ [39]. 

The photothermal performance can be compared with that of other Au morphologies. The efficiency of Au-nanoaggregates has been compared with that of Au-nanoshells (≈150 nm) and Au-nanorods (10 nm × 40 nm) with similar Au concentrations (30 mg·L^−1^). The results show that photothermal efficiency of hybrid nanoaggregates is inferior compared to that of the nanorods and nanoshells, but it can be improved through appropriate design and synthetic methodology.

Thermal stability studies show that nanoaggregates are thermally stable as compared to nanoshells and nanorods as their absorption decreases NIR irradiation. The reason can be the silica coating which prevents shape collapse whereas the non-coated nanoshells and nanorods lose their morphology due to melting and diffusion [40].

Although *in vitro* studies prove the potential of certain materials, the *in vivo* conditions are still difficult to reproduce and multiple variations may occur. *In vivo* tumor ablation requires a tissue temperature of around 48–50 °C for successful operation. Au-aggregates have been investigated *in vivo* and their pharmacokinetics depend on the response of tissues to heat. The surface biofunctionalization is ensured by the silica coating which contributes towards the hydrophilicity. Better accumulation of nanoaggregates at the target site can be attributed to the super-paramagnetic properties which can be influenced by an applied external field.

## 3. Luminescent Gold Speckled Silica (GSS) Nanoparticles

Another aspect of photothermal therapy is the target delivery of the synthesized bionanoprobes to tumor tissues with reduced dosage and subsequent removal from the body [41]. Silica and gold (considered as GRAS materials)-based materials like luminescent Au-speckled-SiO_2_ (GSS) nanoparticles (1–5 nm, irregular shaped, randomly-present nanodomains) were developed and functionalized in this regard. Their fluorescence properties allows selective *in vitro* and *in vivo* PTT operation. The GSS NPs are fabricated in a one-pot water-in-oil (w/o) microemulsion process and the aggregation depends on the concentration of gold which may cause microemulsion instability at higher concentrations. The features of GSS NPs are the size modifications possible, and the presence of fluorescence and absorption in the visible-NIR region [27]. 

The photothermal efficiency of GSS NPs was compared with pegylated GSS nanoparticles and an increase of 11 °C was reported. On comparison with Au-nanoshells (40 nm), GSS NPs show an extinction maximum at 530 nm (*vs.* a maximum extinction peak at 644 nm for Au nanoshells). The difference is attributed to the aggregation to which the nanoshells are prone [42].

**Figure 2:**
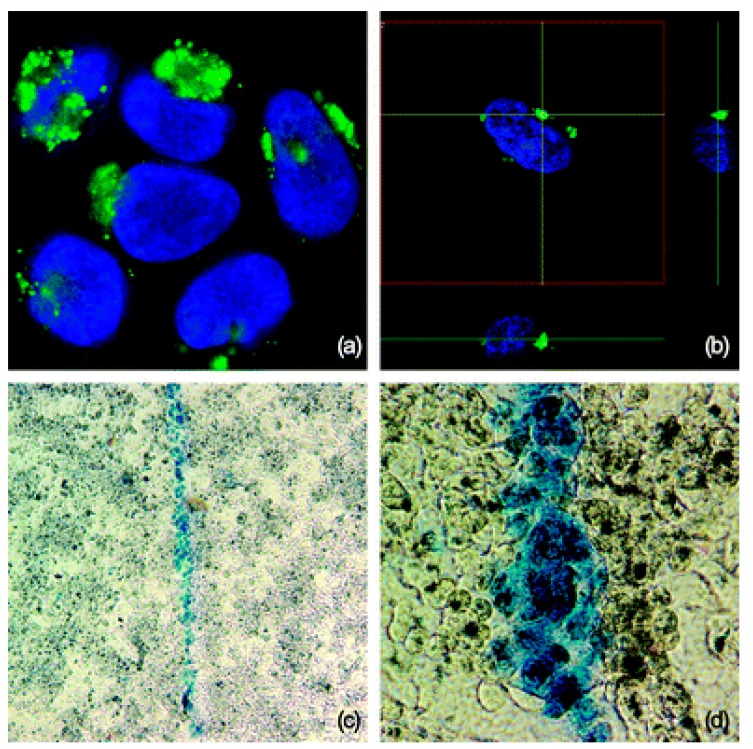
(**a**) Representative confocal microscope picture of lung A549 cells labeled with the FITC doped GSS nanoparticles showing the presence of nanoparticles (green) near the nucleus (blue—stained with Hoechst). (**b**) A z-position cross section showing the localization of GSS nanoparticles adjacent to the nuclear boundary. (**c**) Trypan blue stained dead cells as ablated selectively along the path of the NIR laser and unharmed surrounding cells. (**d**) Higher magnification of trypan blue stained dead cells (reproduced from [38] with permission from The Royal Society of Chemistry).

*In vitro* photothermal therapy of human A549 lung carcinoma cells by using GSS NPs coupled with NIR laser irradiation (785 nm) shows destruction of cancer cells. Without NPs, the laser energy induces no damage to the cells, even after continued exposure. With localized doped GSS NP accumulation, the laser induces a level of heat energy which is sufficient to cause cell death. The cell viability tests performed with trypan blue confirm the cellular damage and apoptosis (Figure 2). The idea is based on discontinuous gold deposits which result in wide NIR absorption, however the use of polyethylene glycol (PEG) in combination with GSS NPs is still not well established. 

## 4. Theranostic Self-Assembly Structured Gold Nanoparticles

Silica is useful, but its hydrophilicity may act negatively as it can bind with biological molecules and consequently damage normal tissues [43]. Therefore, polymers can be another option to fabricate Au-based nanomaterials for PTT. Amphiphilic mixed polymer grafted gold nanoparticles (Au NPs) are designed with inter-particle plasmonic coupling for tuning to the NIR region. Adopting a thiolation method, Au NPs (14 nm) are self-assembled using biodegradable amphiphilic brushes of hydrophilic poly 2-(2-methoxyethoxy)ethyl methacrylate (PMEO_2_MA) and hydrophobic poly(ε-caprolactone) (PCL). Compound micelles (LCM) are prepared by increasing the polymer chain ratio and this contributes to the formation of dense structures which cause a red-shift (520 nm to 830 nm). These compact polymeric Au NPs can be considered equivalent to block copolymers due to their self-assembly into microstructures.

During the synthesis of polymeric Au NPs, hydrophobic PCL chains cover the surface which pulls particles together and results in the distance reduction required for absorption at longer wavelengths. On the other hand, hydrophilic PMEO_2_MA stabilizes the assemblies in aqueous media and causes steric hindrance by reducing the distance between nanoparticles. Therefore combinations of polymers reduce the steric hindrance caused by PMEO_2_MA. The refined thin layer of PMEO_2_MA provides no hurdle to hydrophobic interactions between vesicles to produce LCM. As a result gold assemblies (GAs) are fabricated with alternative ratios of both polymers with different inter-particle distances. In this way, the GA7 displays efficient photothermal ability in which Au NPs are densely packed and allow a red-shift at 830 nm in the NIR region causing an increase of temperature (23 °C). The thermal stability of GAs depends on the concentration of components. An *in vivo* study using esterase determined the hydrolysis of GA7, however the complexity of the human body may alter the stated stability after irradiation. 

**Figure 3:**
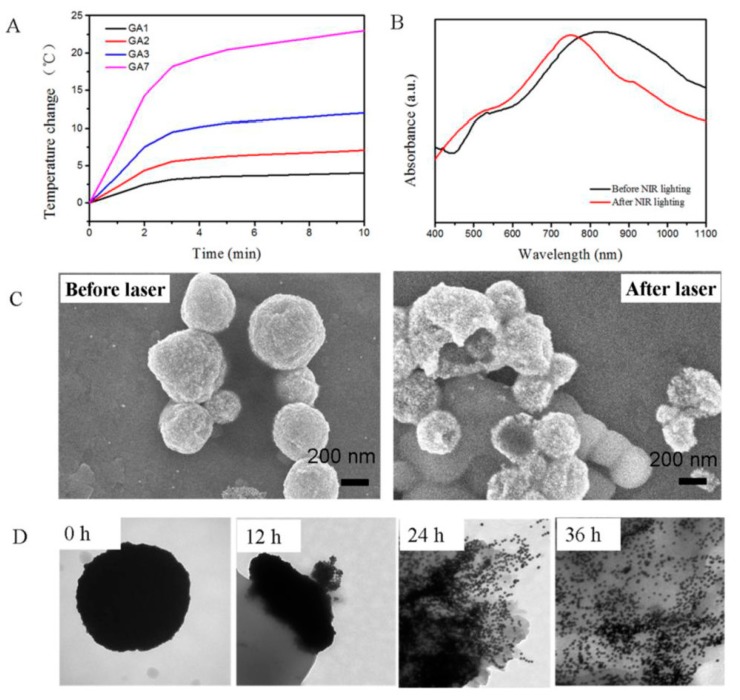
(**A**) The temperature rise curves of aqueous solutions of GAs. (**B**) The UV/Vis/NIR spectra of GA7 before (left) and after (right) NIR illumination. (**C**) SEM images of GA7 before and after NIR illumination. (**D**) TEM images of GA7 before and after incubation with esterase solution at 12 h, 24 h and 36 h respectively (reproduced from [41], an open-access article from Ivyspring International Publisher).

GAs exhibit low toxicity in the absence of laser irradiation. The interaction of GAs with NIR laser and their photothermal ability is confirmed by cell mortality rates (87%) applied to MCF-7 cell lines. The degradation of GA7 at the cellular level before and after the NIR irradiation is different as with no laser energy, only esterase causes hydrolysis of PCL whereas after irradiation complete disintegration into Au NPs is observed (Figure 3).

The *in vivo* photothermal efficiency of GAs determined through MCF-7 tumor-bearing nude mice shows successive cellular damage. There are concerns regarding the removal of GA7, chronic toxicity and complete biosafety [29].

## 5. Photothermal Transducer Au Nanomatryoshkas

Using external coatings via silica shells or polymeric brushes, the removal of nanomaterials from body has limitations. Induced hydrophilicity facilitates the biosorption and delivery of nanomaterials to target site however their prolong permanence can even cause cancer [44,45]. The nanoparticle size controls the tumor uptake and PTT efficiency. Au nanoshells which show high PTT conversion efficiency, non-cytotoxicity and biocompatibility are in clinical trials for their higher absorption cross-sections because of their spherical geometry [46].

However the use of cetyltrimethyl ammonium (CTAB) as surfactant to maintain the shape and negative aggregation, increases its cytotoxicity [47]. Au nano-cages [48] and hollow Au nano-shells (HGNS) made through galvanic replacement reaction have residual Ag and Co which result in cytotoxicity. An integrative-gas-liquid strategy using magnesium NPs is adopted for higher purity [49] however it is yet to be implemented in therapeutics. Au nanoshells with silica coating have failed with the clinically available NIR lasers [50]. The development of clinically translatable NIR-absorbing sub-100 nm Au nanoparticles is challenging [51]. Multilayered Au nanoparticles (Au/SiO_2_/Au) called as nanomatryoshkas has potential as PTT agent however the large scale fabrication has suffered by the lower efficiency of amine functionalization used as precursor for Au/SiO_2_ nanoparticle before terminal Au layer [52]. 

**Figure 4:**
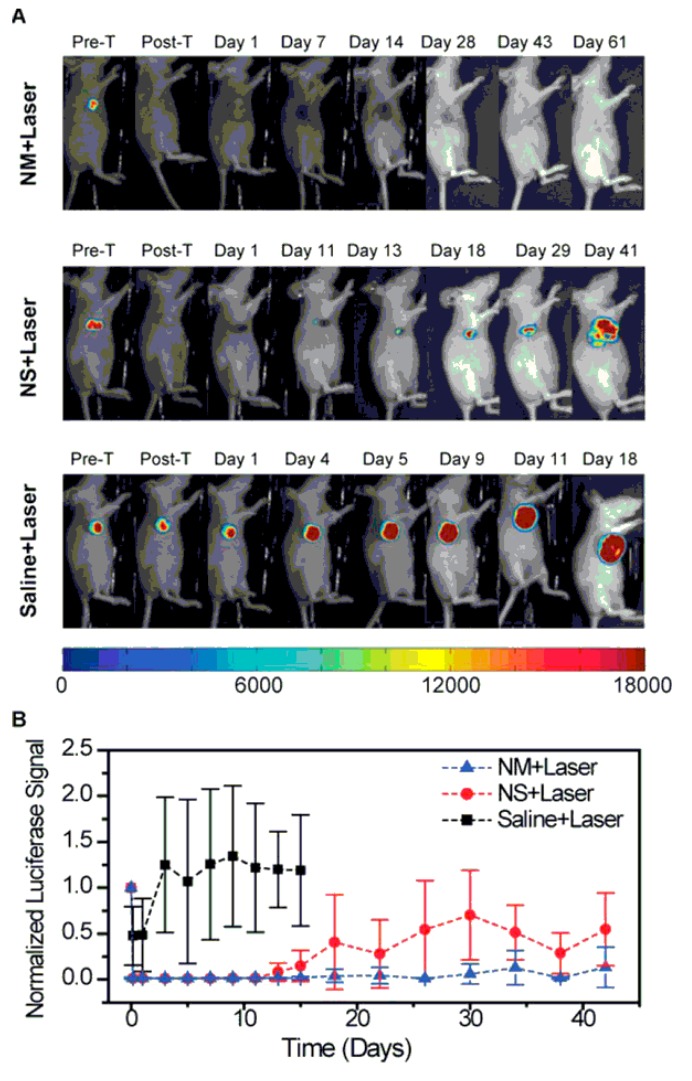
Evaluation of tumor response to photothermal therapy by bioluminescence imaging. The bioluminescence signal is generated only in living cancer cells as a result of luciferase activity. (**A**) Representative mice of each experimental group showing the luciferase activity in the tumor. The mice injected with nanomatryoshkas or nanoshells and treated with laser experienced loss of bioluminescence in the area illuminated by the laser as seen after therapy. Mice were euthanized when tumor volume reached 1,500 mm^3^ or if the tumor persisted at 60 days after treatment. (**B**) Mean luciferase activity in the tumor with standard deviations. The luciferase signal was normalized to the signal before treatment (reprinted with permission from [49]; copyright (2014) American Chemical Society).

Au nanomatryoshkas (Au/SiO_2_/Au, ~90 nm) in comparison to Au nanoshells (~150 nm) as PTT agents using triple negative breast cancer (TNBC) tumors in mice are tested [28]. Difference of absorption efficiency in Au-nanomatryoshkas (77%) and nanoshells (only 15%) determines the performance as PTT agent. In vivo injection of Au nanomatryoshkas and single NIR laser dosage (2 W/cm^2^ for 5 min, 83%) to TNBC tumor-bearing mice show health improvement and complete recovery in less than two months whereas 33% of mice treated with the nanoshells lived within the same period. The better performance of Au nanomatryoshkas resides with reduced size and large absorption cross section as compared to Au nanoshells (Figure 4). 

Previously amine functionalization was done on silica coated gold colloids. The synthetic improvement in Au nanomatryoshkas involving the doping of the SiO_2_ layer with (3-aminopropyl)-triethoxysilane (APTES) enhances the binding of Au colloids (12 nm). The thickness of the silica layer affects the plasmonic resonance essential for NIR absorption. The precursor is first introduced to obtain a thickness of ~16 nm followed by etching through hydrolysis and formation of an Au shell in the last step. This assembly exhibits red-shifts in the plasmon resonance and the efficiency of nanomatryoshkas is enhanced 1.6 times more than the nanoshells at an optical density of 1.

Neutral surface charge prolongs the circulation time in the bloodstream and enhances tumor penetration. Nanomatryoshkas and nanoshells are functionalized with thiolated poly(ethylene glycol) (thiol-PEG) for biocompatibility. Additionally, a smaller size of nanomatryoshkas ensures more tissue uptake of Au content in the tumor (μg of Au per g of tumor) and was found to be ~1.7 times higher for nanomatryoshkas than for the nanoshells. After 5 min of laser irradiation, a higher temperature was expected for the nanomatryoshkas injected group than the nanoshell one, however no statistically significance difference is observed.

## 6. Gold Nanorods (GNRs)

GNRs are more efficient than nanospheres and nanoshells because of their polarization properties. Nanorods act as antennae along their axis and exhibit circular polarization due to random orientations in cells [53]. This helps reach high temperatures in cancer tissues resulting in necrosis or cell death. Certain synthetic routes have been established for GNRs for enhanced light scattering properties like a seedless growth method [54] and a one-pot synthesis using phenols [55]. A comparative study using gold nanorods (GNRs, prepared by a seed-mediated method) as PTT agent shows that GNRs reduce the tumor size and increase the survival rate more than chemotherapy [25]. The NIR light-emitting diode (LED) as optical source works for thermal ablation in combination with GNRs as a therapy. *In vitro* models also show that heat is only observed in the presence of NIR-exposed GNRs. The localized thermal ablation gives selective patterns for GNRs in tumor tissues however laser irradiation in healthy tissues in the absence of GNRs causes negligible damage. The distribution of GNRs faces the problem of non-specific toxicity in normal tissues [56], therefore amphiphilic coatings of polymer-polyethylene glycol (PEG) on nanorods enhance their stability and biocompatibility for cancer therapy. Without functionalization, GNRs cannot infiltrate the blood vessels and therefore their concentration increases in plasma. Biocompatibility of PEG is based on the uncharged hydrophilic groups which show high surface mobility along with the resistance towards protein adhesion and biological attack. It exhibits non-immunogenicity and non-antigenicity [57,58]. Therefore PEGylated GNRs show increased half-life and bio-distribution in *in vivo* models. 

## 7. Thermo-Responsive Polymer Encapsulated Gold Nanorods

Polymeric coatings that induce biocompatibility will be favorable if the polymer can also display thermo-responsive properties. To this end GNRs (Au@SiO_2_) are encapsulated in a thermo- and pH-responsive polymer, poly(N-isopropylacrylamide-co-acrylic acid), as a single nanocomposite. Doxorubicin (Dox) is then loaded onto the nanocomposite to couple chemo- and radio-therapeutics in a single step using electrostatic interactions and attaining a high loading capacity of up to 24% [59]. The thermo-responsive nature of PNIPAM allows a reversible phase transition involving compact hydrophobic globular structure formation at a raised temperature (<32 °C). Secondly the drug (Dox) combines to the nanocomposite via electrostatic absorption and it also helps to reduce the size necessary for penetration into the tissue on irradiation.

External heating of the thermo-responsive polymer facilitates the tissue penetration of the so-called Nanocom-Dox which is tested by coupling a fluorescent dye (IR820) with the nanocomposite. Such a response can be generated by using a NIR laser as a heat source. The increase in temperature of tumors is ~36 °C at the tumor site after injection with Nanocom-Dox. The increase in temperature increases the pore size, which enhance the vascular permeability and shrinks the size of the nanocomposite. As a non-thermo-responsive control, Au@SiO_2_-PEG is tested against Au@SiO_2_-PANIPAM and differences are observed between the nanocomposites, with better performance of Au@SiO_2_−PNIPAM due to its reduced size. However, after the irradiation is cut off, the size of nanocomposites increases and they are not able to cross the vascular system therefore they may reside in the body and may affect the normal tissues due to the cytotoxic effects of PNIPAM [60]. 

## 8. Conclusions and Future Perspectives

NIR technology has been improved and shows broad perspectives in life sciences. Coupled to nanotechnology, NIR-based photothermal therapy is a major development area. Gold nanoparticles have a major impact in plasmonic photothermal therapy because of their simpler synthesis, easy surface functionalization and diverse morphologies. Nevertheless the Au-based nanomaterials have failed in clinical trials as PTT agents. The problems involve critical parameters like how Au-nanomaterials behave at the cellular level, compatible biodistribution, the detoxification and clearance of the nanoparticles. In the future, *in vivo* research studies will become customary to explore the PTT efficiency of Au nanomaterials. Same nanomaterials may not work for all types of cancers because of the nature of the tissue involved. Therefore in-depth analysis and compatibility of NPs is crucial. Similarly status of damage to healthy tissue must be considered as it may hinder the use of nanomaterials at the clinical level.
